# A Promising Point‐of‐Care Testing Strategy: Ultrasmooth Gold Nanogroove Arrays Biosensor Combined with Initial Rate Analysis

**DOI:** 10.1002/advs.202503056

**Published:** 2025-07-22

**Authors:** Yi Liu, Shan Xing, Zhan Si, Kai He, Manchun Zheng, Yang Shen, Chongjun Jin

**Affiliations:** ^1^ State Key Laboratory of Optoelectronic Materials and Technologies, School of Materials Science and Engineering Sun Yat‐Sen University No.132, East Outer Ring Road, Guangzhou Higher Education Mega Center Guangzhou Guangdong 510006 China; ^2^ Department of Clinical Laboratory State Key Laboratory of Oncology in South China, Guangdong Provincial Clinical Research Center for Cancer, Sun Yat‐sen University Cancer Center 651 Dongfeng East Road Guangzhou Guangdong 510060 China

**Keywords:** initial rate analysis (IRA), plasmonic biosensors, tumor marker detection, ultrasmooth gold nanogroove arrays (UGNA)

## Abstract

Nowadays, plasmonic biosensors have achieved the limit of detection (LOD) far beyond the clinical or professional standards in the sensing field through material and structural optimizations. However, it remains predominantly a research tool due to the challenge to simultaneously achieve mass production, ultrasensitive and rapid detection, high‐reproducibility, and ease of integration, which are extremely desired in point‐of‐care testing (POCT)‐based commercial products. Here, a label‐free, ultrasensitive, and rapid assay protocol is described for quantitative protein detections in serum samples, which integrates an ultrasmooth gold nanogroove arrays (UGNA) biosensor with an initial rate analysis (IRA) method. i) Compared with the existing plasmonic biosensors, miscellaneous‐protein‐mixed scheme substantially accelerates the binding kinetics of analytes by passivation of all channel inner surfaces. Combined with the IRA method, a wide linear range of AFP detection (1–10^4^ ng mL^−1^) is obtained, and an enhancement factor of ≈160‐fold in detection time (≈70 s) is achieved for an analyte with ultralow concentration. ii) UGNA can achieve an extremely high surface figure of merit (FOM_surf_) under normal incidence, which is immensely useful for miniaturization and multiplexing. iii) UGNA are fabricated using a high‐reproducibility template‐stripping technique, which potentially enables low‐cost mass production. These unique advantages suggest that the biosensor system and analysis method have tremendous potential in POCT biosensing devices.

## Graphical abstract

This work reports a nanoplasmonic biosensing platform with exceptional surface sensitivity (0.618 nm nm^−1^, Δn = 0.117 nmol) and narrow linewidth (≈20 nm) under normal incidence. By integrating a miscellaneous‐protein strategy with initial rate analysis (IRA), this sensor can detect 1 ng mL^−1^ of AFP in serum with a broad linear range (1–10^4^ ng mL^−1^) within 1 min, surpassing commercial chemiluminescence systems.

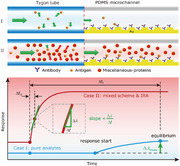

## 1 Introduction

Plasmonic biosensors are extensively used in medical diagnosis, food safety regulation, and environmental monitoring.^[^ ^1^, ^2^, ^3^, ^4^ ^]^ Thanks to the subwavelength light confinement and enhancement near the metallic surface, surface plasmon resonances (SPRs) can generate remarkable optical responses to the minuscule changes of local refractive index (RI) near the sensor surface induced by the biomolecular binding events.^[^ ^5^, ^6^, ^7^, ^8^ ^]^ The molecular interactions occurring on the sensor surface can be optically monitored in real‐time via various interrogation methods (for example, angular, wavelength, intensity, or phase interrogations).^[^ ^9^, ^10^, ^11^, ^12^ ^]^ This unique feature enables the plasmonic biosensor to be an indispensable tool for real‐time and label‐free analysis in biomedical diagnostics. To date, considerable efforts in this field have been devoted to pushing the LOD of the plasmonic biosensors beyond of the current clinical devices.^[^ ^13^ ^]^ These strategies include the multiscale aspects: First, at the mesoscopic level (micro/nano‐structural level), structural optimizations of the plasmonic architectures in terms of surface sensitivity and FOM have been extensively explored.^[^ ^14^, ^15^, ^16^ ^]^ The key issue involved is how to synchronically achieve more concentrated local fields at the sharp resonances (i.e., with narrower full width at half maximum, FWHM) by suppressing their internal or/and radiative losses.^[^ ^17^, ^18^ ^]^ Second, at the microscopic level (biomolecular level), surface modifications of the sensor chips have been demonstrated to significantly improve the efficiencies of the receptor immobilizations and the affinity bindings between the analytes and receptors.^[^ ^19^, ^20^, ^21^ ^]^ In addition, analyte concentration and signal amplification techniques can substantially enhance the sensitivities.^[^ ^22^, ^23^, ^24^, ^25^, ^26^ ^]^ Third, at the macroscopic level (device level), the performance of the sensor system can be further boosted by utilizing unconventional interrogation methods (e.g., phase interrogation) with a higher instrumental resolution and lower noise level.^[^ ^20^ ^]^ Despite these impressive progress in boosting the LODs, the requirements for detection indicators in clinical testing are far beyond LOD. Table S1 (Supporting Information) lists the key performance metrics for a series of plasmonic biomolecular detection platforms.^[^ ^27^, ^28^, ^29^, ^30^, ^31^, ^32^, ^33^, ^34^, ^35^, ^36^, ^37^, ^38^, ^39^, ^40^ ^]^ From a practical perspective, a sensor has clinical utility only if an accurate analyte quantitation is accomplished within an acceptable time frame (such as a few minutes). Unfortunately, due to the limitation by mass transport in delivering biomolecules from the bulk solution into the capture volume of sensors, the detection time is often in an unacceptable range, especially at an ultralow analyte's concentration.^[^ ^41^, ^42^ ^]^ To reduce the response time, diverse strategies have been proposed. For example, flow‐through nanohole arrays can offer ≈a 10‐fold improvement in response time over the established flow‐over sensors for typical binding kinetics and analytes.^[^ ^43^ ^]^ In addition, chaotic mixing in the sample flow by a 3D serpentine microchannel has also been shown to provide an enhancement factor of 2 in the analyte binding rate.^[^ ^44^ ^]^ Besides, based on the electrokinetic effects (electrophoresis, dielectrophoresis, and electrothermoplasmonic effects), the target molecules can be transported more efficiently to the surface of plasmonic sensing sensor by applying external electric fields and/or creating temperature gradients.^[^ ^45^, ^46^, ^47^ ^]^ However, these strategies need either more complex microfluidic systems (such as double‐layered microchannels or structured microchannels) or additional electrical and heating elements, which is unfavorable to the miniaturization of the sensing system.

In this work, we propose and demonstrate an ultrasensitive and rapid plasmon biosensing platform for quantitative protein detection in serum samples. On the one hand, we developed an ultrasmooth gold nanogroove arrays (UGNA) nanophotonics biosensor chip with a narrow FWHM of ≈20 nm. It exhibited a high surface thickness sensitivity (*S*_surf_) of 0.618 nm nm^−1^ for detecting a molecular layer (RI = 1.45) in water (*n* = 1.333). Such an excellent sensing performance of the UGNA was achieved under normal illumination, which is beneficial for miniaturization and multiplexing.^[^ ^5^ ^]^ On the other hand, by implementing a miscellaneous‐protein‐mixed scheme, the binding kinetics of the analyte was significantly accelerated. More importantly, combined with the IRA approach, the detection time for the miscellaneous‐protein‐mixed alpha‐fetoprotein (AFP) solution with an analyte concentration of 1 ng mL^−1^ can be further reduced to within 70 s. This is ≈160 times faster than the detection time for the AFP with same concentration in phosphate buffer solution (PBS). The linear detection range over 1–10^4^ ng mL^−1^ was achieved, superior to mainstream commercial chemiluminescence analyzers. We further implemented this platform for clinical sample detection, and the measured results were reasonably consistent with those obtained from commercial electrochemiluminescence detection equipment (Roche Cobas 8000 e801). In addition, the UGNAs were fabricated using the template‐stripped method, potentially enabling cost‐effective mass production and chip repeatability. These advantages pave the way toward low‐cost, fast, sensitive, reproducible, and miniaturized biosensors for POCT.

## 2 Design of a Protein Detection System by Integrating UGNA Biosensor and IRA Method

**Figure 1:**
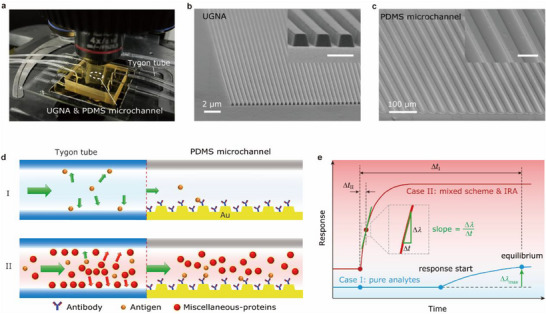
Label‐free detection of antigens with a UGNA biosensor. a) Photograph of a UGNA biosensor platform. b) Side‐view SEM image of a UGNA. The inset shows its local magnification. The scale bar is 500 nm. c) Side‐view SEM image of the PDMS microchannel. The inset shows its local magnification. The scale bar is 50 µm. d) Schematics showing the analyte depletion effect caused by the adsorptions on the inner walls of the tubes in the microfluidic system. I and II represent distinct antigens binding efficiencies on the sensor surface for the two cases: I) a solution containing the target antigen in PBS, and II) an antigen solution mixed with extra miscellaneous proteins. e) Time‐response curves of the antigen detections for the two cases in d), where the blue and red curves denote cases I and II, respectively. The introduction of the miscellaneous proteins and the IRA method synergistically shortens the detection time (Δ*t*_I_ ≫Δ*t*_II_).

A miniaturized label‐free biosensor platform (**Figure** **1**a) was developed by assembling a microfluidic system on top of the UGNA biochip (see Methods). The basic sensing unit (indicated by the white circles in Figure 1a), namely UGNA, consists of an array of periodic gold nanogrooves (lattice constant *a* = 500 nm, groove depth *d* = 245 nm, top and bottom widths of the groove *w*_1_ = 280 nm and *w*_2_ = 130 nm), as shown in Figure 1b. The inner side of the microchannel features a periodic micro‐grating array (periodicity *p* = 40 µm, stripes height *h* = 5 µm, stripes width *w* = 10 µm) oriented perpendicular to the flow path. Additionally, the micro‐grating is decorated with randomly distributed microdisks with diameters ranging from 1 to 3 µm. These combined structural elements enhance analyte transport efficiency and reduce background noise caused by the Fabry‐Pérot effect during detection. The details on the fabrication and integration processes of the UGNAs and PDMS microchannels can be found in Methods and Figures S1–S3 (Supporting Information).

In addition, we developed a miscellaneous‐protein‐mixed scheme combined with the IRA method to reduce total detection time. Generally, after injecting analytes in PBS into a microfluidic system, the sensor response will undergo a rapid growth at first and reach a steady state after a certain period. Unfortunately, as the analyte concentration decreases, the time to reach equilibrium will be exceptionally increased. For example, it takes more than 2 h to reach equilibrium for 1 ng mL^−1^ AFP antigen detection with a flow rate of 1 mL h^−1^ (Figure S4, Supporting Information). Besides, we found that the sensor has an unacceptably long response delay (≈5000 s) for the AFP concentration of 1 ng mL^−1^ after the sample injection (blue curve in Figure 1e; Figure S4, Supporting Information). Such a response delay is probably due to the non‐specific binding of the antigen molecules to the inner walls of the inlet tube. This results in an undesired antigen depletion in advance. So that only few antigen molecules can enter the microchannel and bind to the sensor surface, as schematically shown in case I in Figure 1d. To avoid response delays at low‐concentrations, high‐concentration miscellaneous proteins are introduced into the antigen solution to block the inner walls of the inlet tube, preventing the non‐specific antigen binding (case II, Figure 1d). This approach significantly enhances antigen‐antibody binding kinetics, as demonstrated by the red curve in Figure 1e. To further reduce the total detection time, we use the IRA method to determine the antigen concentration.^[^ ^48^ ^]^ When the analyte solution is continuously replenished to keep its bulk concentration constant, the reaction between the immobilized receptor and the analyte in the solution can be considered to follow pseudo first‐order kinetics. In this situation, the rate of the sensor response d*R*/d*t* can be written as

(1) $$\frac{dR\left(t\right)}{dt}=k_{a}C\left(R_{max}-R\left(t\right)\right)-k_{d}R\left(t\right)$$

where *C* is the analyte concentration in solution, *R*_max_ is the maximum sensor response, *R*(*t*) is a sensor response at time *t*, *k*_a_ and *k*_d_ are the association and dissociation rates, respectively. Here, we assume that the dissociation can be neglected (*k_d_* ≈ 0) in the initial binding phase, the sensing surface has high binding capacity (*R_max_* ≫ *R*(*t*)), and the mass transfer limiting condition will be established. We can construct a linear calibration curve of lg(Δ*R*/Δ*t*) vs. lg(*C*) in this situation. Our assay protocol benefits from enhanced kinetics (eliminating response delays and saturation requirements), allowing substantial time savings. The total detection time for low analyte concentrations is shortened by at least 100 times compared to conventional methods.

## 3 Optimization of UGNAs

**Figure 2:**
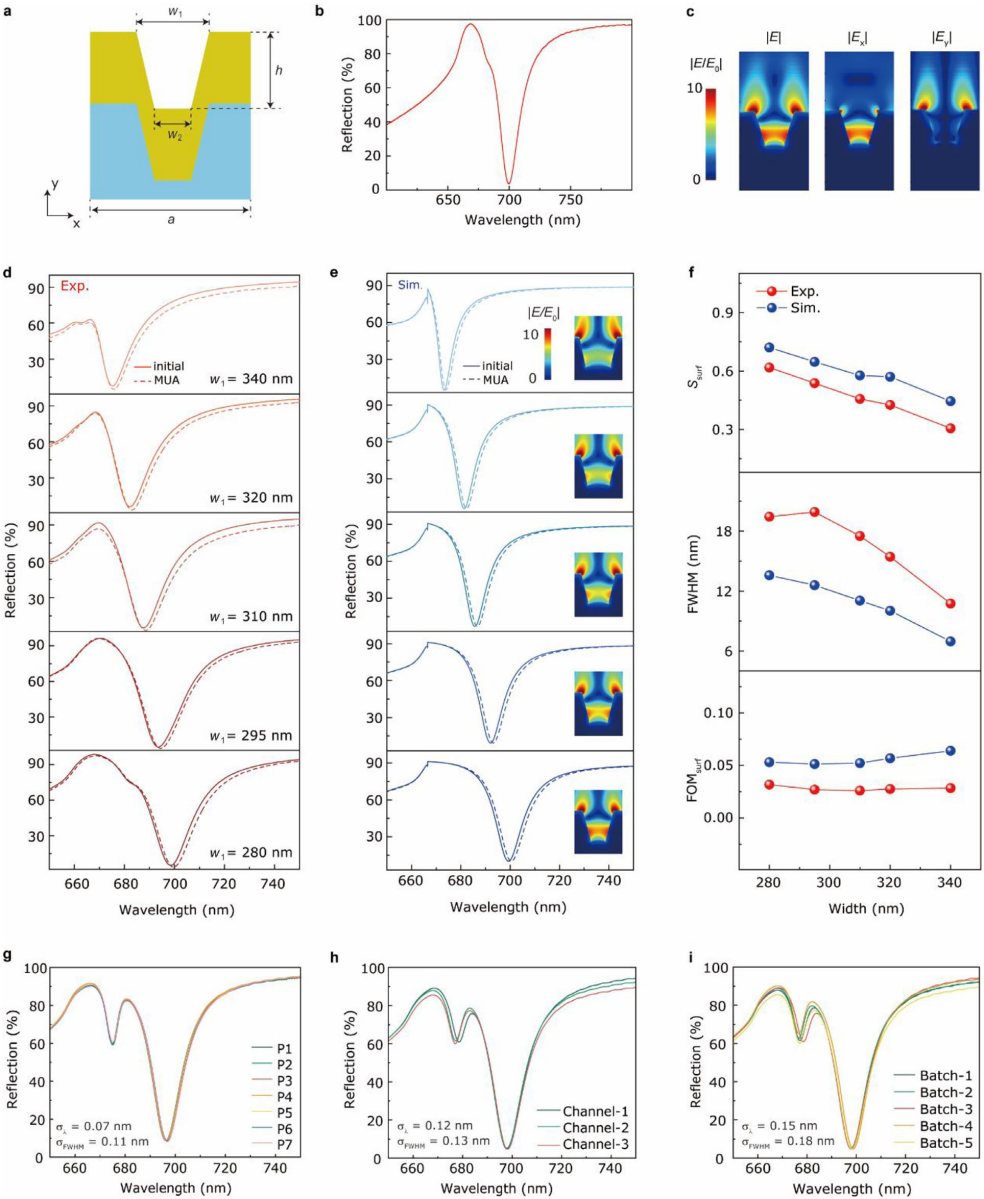
Structural optimization of UGNAs. a) Schematics of a unit cell of the UGNA. b) Experimental zero‐order reflectance spectrum of a UGNA (*a* = 500 nm, *d* = 245 nm, *w*_1_ = 280 nm, and *w*_2_ = 130 nm) at normal incidence. c) Simulated distributions of the electric field intensities | ***E*** |, | ***E**_x_* |, and | ***E**_y_* | in the *x*‐*y* plane at the wavelength of 697.8 nm, respectively. d),e) Experimental and simulated zero‐order reflectance spectra of a series of UGNAs with various top widths of the grooves. The solid and dashed curves denote the untreated UGNAs and the UGNAs adsorbed by the SAM of MUA, respectively. Insets in Figure 2e are the corresponding electric field intensity distributions at the resonant dips. f) Comparison of the simulated (blue) and experimental (red) *S*_surf_, FWHM, and FOM_surf_ as functions of the top widths of the grooves. g) Point‐to‐point reproducibility from the different points in a single UGNA. h) Channel‐to‐channel reproducibility from the different UGNAs but in the same chip. i) Batch‐to‐batch reproducibility from the different chips in various duplications.

The *S*_surf_ and FWHM are two critical performance metrics for evaluating the intrinsic sensing capability of evanescent‐field‐based biosensors. Here, the *S*_surf_ is defined as the ratio of the change in sensor response (e.g., wavelength shift, Δ*λ*) to the thickness variation Δ*h* in a conformal molecular layer with a specific RI (*n*_mol_):

(2) $$S_{\mathrm{surf}}=\frac{\Delta \lambda }{\Delta h}=\frac{S_{\mathrm{bulk}}\times \left(n_{\mathrm{mol}}-n_{\mathrm{bg}}\right)\times \left(1-e^{-2h/l_{d}}\right)}{\Delta h}$$

where *S*_bulk_ is the bulk sensitivity (Δ*λ*/Δ*n*), *n*_mol,_ and *n*_bg_ are the refractive indices of the molecular layer and background environment (e.g., air or solutions), respectively, and *l*_d_ is the evanescent field decay length. From Equation (2), to achieve the highest *S*_surf_, one should generate an extremely localized field with minimum *l*_d_. In the view of practical sensing, although the sensor response is ultimately dependent on the spectral shift Δ*λ*, a narrow FWHM is still highly desired. This is for a sharper peak or dip, and the resonant wavelength will be more precisely determined, leading to a lower noise level. Unfortunately, a highly localized field profile is generally incompatible with a narrow resonant linewidth. Thus, considering a trade‐off between *S*_surf_ and FWHM, we optimized the sensing performance of UGNAs in terms of FOM_surf_, which is defined as *S*_surf_ /FWHM (an analogue to FOM in bulk sensing). The geometry of the UGNA can be fully characterized by its lattice *a*, groove depth *d*, and top and bottom groove widths *w*_1_ and *w*_2_, as schematically depicted in **Figure** **2**a. Figure 2b shows a typical reflection spectrum of a UGNA (*a* = 500 nm, *d* = 245 nm, *w*_1_ = 280 nm, and *w*_2_ = 130 nm) in water which features an extremely narrow resonant dip located at the wavelength of ≈700 nm. Figure 2c shows the simulated electric field intensity distributions of the UGNA at this dip, suggesting that such a sharp resonant dip originates from the coupling between the Fabry‐Pérot mode (|*E*_x_|, Figure 2c) and Wood's anomaly mode (|*E*_y_|, Figure 2c).^[^ ^17^ ^]^ Here, we simulated the lattice‐, depth‐, and width‐modulated reflection spectra of the UGNAs (Figure S5, Supporting Information) by using the finite‐difference time‐domain (FDTD) method. The details on the FDTD numerical simulations can be found in Methods. These simulated results reveal that at the fixed lattice and depth (*a* = 500 nm, *d* = 245 nm), the critical coupling can be established (i.e., achieve nearly 0% reflectance at the resonant dips) over a broad modulation range of groove widths (Figure S5c, Supporting Information). Thus, we further investigated the effects of the groove width on *S*_surf_ and FWHM under critical coupling conditions. To evaluate the width‐dependent surface sensing capability, a self‐assembly monolayer (SAM) of 11‐mercaptoundecanoic acid (MUA) (*h* = 1.7 nm, *n*_mol_ = 1.45) was utilized due to its highly uniform surface coverage and well‐defined chemical functionality.^[^ ^49^ ^]^ We fabricated a set of UGNAs with top width ranging from 280 to 340 nm. The other structural parameters and the corresponding SEM images are summarized in Table S2 and Figure S6 (Supporting Information). Figure 2d,e shows the experimental and simulated width‐modulated reflection spectra of the bare and modified UGNAs with a monolayer of MUA. The measured and simulated results exhibit a similar evolution trend of *S*_surf_, FWHM (Figure 2f). As the groove width is reduced, the *S*_surf_ of the UGNA is improved, but the FWHM is broadened simultaneously. The experimental *S*_surf_ is smaller than the simulating prediction, which is caused by the molecule is not being closely assembled at the surface of UGNA in the measurement. The broaden experimental FWHM is induced by fact that the inherent loss of the gold film is higher than the perfect gold crystal film. To further elaborate the physical mechanism behind these trends, a near‐field optical picture is presented (insets in Figure 2e). For the narrower groove, a more concentrated electric field localized at the apexes of the groove opening and a stronger field inside the groove are observed, suggesting a higher *S*_surf_ together with larger radiative and internal losses (i.e., broader FWHM). Considering both *S*_surf_ and FWHM, the FOM_surf_ values remain relatively constant within the range of *w*_1_ from 280 to 340 nm. Therefore, *w*_1_ = 280 nm was selected, at which *S*_surf_ reaches the highest value of 0.618 nm nm^−1^. Subsequently, we prepared a batch of UGNAs with the parameters of *a* = 500 nm, *h* = 245 nm, *w*_1_ = 280 nm, and *w*_2_ = 130 nm using the templated stripped method.^[^ ^17^ ^]^ Figure 2g–i depicts the point‐to‐point, channel‐to‐channel, and batch‐to‐batch reproducibility of the UGNAs, respectively. Here, the “point‐to‐point” refers to the randomly chosen points on the same UGNA, the “channel‐to‐channel” refers to the different UGNAs from the same chip, and the “batch‐to‐batch” refers to the different chips duplicated from the same silicon template. Tiny standard deviations of the resonance wavelengths and FWHMs were achieved (*σ_λ_* = 0.07 nm and *σ*_FWHM_ = 0.11 nm for point‐to‐point reproducibility; *σ_λ_* = 0.12 nm and *σ*_FWHM_ = 0.13 nm for channel‐to‐channel reproducibility; *σ_λ_* = 0.15 nm and *σ*_FWHM_ = 0.18 nm for batch‐to‐batch reproducibility).

## 4 Tumor Marker Detection in PBS

We chose AFP as an analyte to demonstrate the applications of our UGNA‐based sensing platform in tumor biomarker detection. AFP is the widely used tumor biomarker for hepatocellular carcinoma (HCC) and germ cell tumors.^[^ ^50^ ^]^ Serum AFP concentrations exhibit dynamic pathological characteristics from below 20 ng mL^−1^ in healthy and chronic liver disease patients to over 10,000 ng mL^−1^ in advanced HCC. These levels correlate with disease progression, therapeutic response, and prognosis.^[^ ^51^, ^52^ ^]^ The extraordinary dynamic range from “nanogram” to “microgram” levels, directly reflects tumor aggressiveness and volume, thus necessitating a broad linear detection range to accurately capture the full pathological spectrum of HCC.

**Figure 3:**
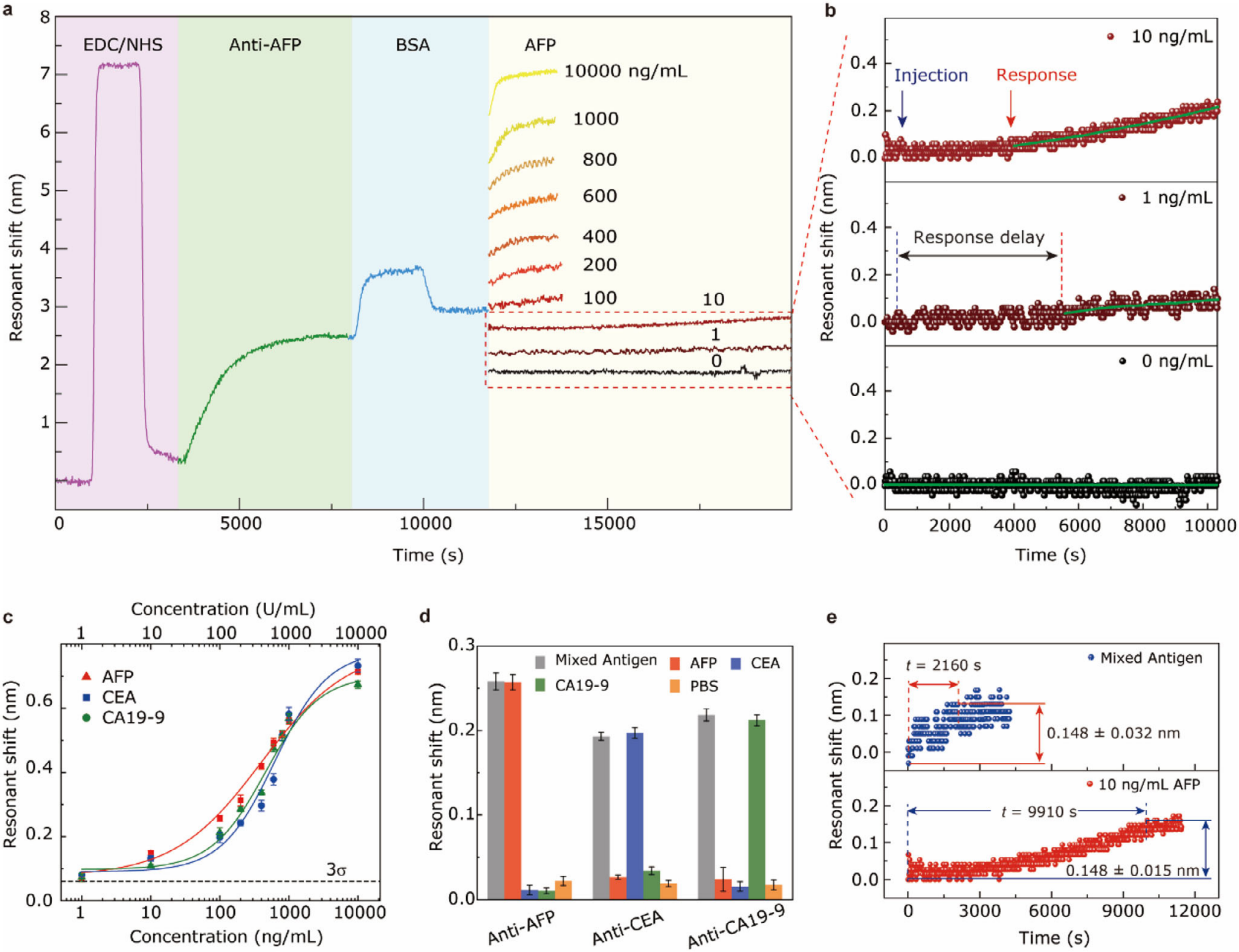
UGNA biosensor modification and measurement in PBS buffer. a) Real‐time responses of the multiple sensing channels for detecting the AFP antigen with various concentrations. The chip was modified with 100 mg mL^−1^ anti‐AFP. b) Enlarged view of the real‐time response curves for 0, 1, and 10 ng mL^−1^ AFP antigens, respectively. c) Resonant shift (Δλ) as functions of the concentrations for three kinds of antigens, including AFP (red), CEA (blue), and CA19–9 (green). The error bars represent the standard deviations calculated from three data points measured at each concentration. d) Specificity analysis of a UGNA biosensor for antigen detection. Among the mixed antigens were 100 ng mL^−1^ AFP, 100 ng mL^−1^ CEA, and 100 U mL^−1^ CA19‐9. e) Comparison of the response between 10 ng mL^−1^ AFP antigen and a mixture of antigens (including 10 ng mL^−1^ AFP, 10 ng mL^−1^ CEA, and 10 U mL^−1^ CA19‐9).

For quantitative AFP detection, the UGNA surface was first functionalized with a monolayer of MUA, which is confirmed by the corresponding Fourier transform infrared (FTIR) spectra (Figure S7, Supporting Information). Subsequently, we assembled a microfluidic chip by bonding a PDMS slab with microchannels to the surface‐functionalized UGNA under oxygen‐plasma treatment. Next, we performed a set of operations to modify the UGNA surface by sequential injection of different reagents with a flow rate of 1 mL h^−1^, including carboxyl activation, antibody immobilization, and bovine serum albumin (BSA) blocking. Finally, a series of AFP solutions in PBS with various concentrations (1 to 10^4^ ng mL^−1^) were injected into the multiple sensing channels to determine the detection sensitivity and kinetics of the UGNAs. A real‐time response in each step was monitored by tracking the spectral shifts (**Figure** **3**a) on a homemade micro‐area reflection measurement system (Figure S8, Supporting Information). A rapid spectral shift of ≈7 nm was observed when carboxyl activation reagents (EDC/NHS) was injected. This phenomenon is primarily attributed to the alteration in the bulk refractive index resulting from the substitution of the buffer from PBS (*n* = 1.334) to MES (*n* = 1.340). After Anti‐AFP modification and BSA blocking, the spectral responses exhibited an initial rapid shift followed by a gradual stabilization, indicating adsorption saturation. In the final AFP antigen detection, as the concentration of AFP antigen increased, the shift of the spectrum also became progressively larger. For high concentrations of AFP (i.e., 200 to 10000 ng mL^−1^), the response was initiated instantaneously once the AFP solution flowed through the UGNA, and gradually transited to an equilibrium state level, offering relatively large responses (Δ*λ * =  0.32−0.72 nm) and fast binding kinetics (*t*_response_ = 1200–2000 s).

However, for low concentrations of AFP (i.e., 1 and 10 ng mL^−1^), the response is remarkably slowed down, which manifests primarily as an extended delay and a low and constant response rate (Figure 3b). These response delays are probably attributed to the non‐specific binding of the antigen molecules to the inner surfaces of the inlet tubes. From the Δ*λ*−*C* curve of AFP presented in Figure 3c, the smallest discernible concentration above the 3*σ* baseline was deemed to be 1 ng mL^−1^. A broad dynamic range was determined to be 1 to 10^4^ ng mL^−1^. Technically, the upper limit of the linear range must balance clinical utility and detection reliability. Restricting the upper threshold to <100 ng mL^−1^ would require repeated dilutions for high‐concentration samples, increasing laboratory complexity, costs, and potential errors from miscalculations or operational variability. By extending the linear range to 10^4^ ng mL^−1^, our method enables “single‐test” for >95% of clinical samples (including extreme highs), significantly outperforming commercial systems (e.g., Roche Cobas e801: 0.908–1210 ng mL^−1^). This design ensures continuous quantification accuracy across low‐concentration gray zones (e.g., 7–20 ng mL^−1^ in active chronic liver disease) and extreme pathological elevations.

In addition, the averaged coefficient of variation (CV) for all measured concentrations was relatively low (3.6%), indicating a highly reproducible assay. To further verify the universality of our UGNA‐based protein detection platform, we also detected two other tumor markers, namely, carcinoembryonic antigen (CEA, blue curve in Figure 3c) and carbohydrate 19‐9 antigen (CA19‐9, green curve in Figure 3c) in PBS. The detection limits for CEA and CA19‐9 were determined to be 1 ng mL^−1^ and 1 U mL^−1^, with average CVs of 5.2% and 3.8%, respectively. To further assess the specificity of AFP detection, we prepared a mixed antigen solution consisting of 100 ng mL^−1^ AFP, 100 ng mL^−1^ CEA, and 100 U mL^−1^ CA19‐9. AFP served as the target analyte, while the other tumor markers (CEA and CA19‐9) were used as controls. As shown in Figure 3d, the control samples showed negligible response, whereas the mixed antigen solution and AFP solution produced similar response levels. This indicates that the observed response was exclusively caused by the specific interaction between the AFP antigen and the immobilized anti‐AFP. Similarly, the specificity of CEA and CA19‐9 detections was also assessed using the same method.

Surprisingly, both the target antigen and the mixed antigen generated the same response level but exhibited significant kinetic differences at low concentration. As shown in Figure 3e, the 10 ng mL^−1^ AFP solution took ≈9910 s to reach equilibrium, whereas the mixed antigen solution needed only ≈2160 s. The faster kinetics for the mixed antigen can be explained by the fact that the more miscellaneous proteins from the mixed antigen were nonspecifically adsorbed on the channel inner surfaces, suppressing the nonspecific adsorption of AFP. This suggests that one can greatly enhance the antigen‐antibody binding kinetics by a complete channel surface passivation using miscellaneous proteins with higher concentrations.

## 5 Faster Tumor Marker Detection Enhanced by Miscellaneous‐Proteins‐Mixed Scheme Combined with IRA Method

**Figure 4:**
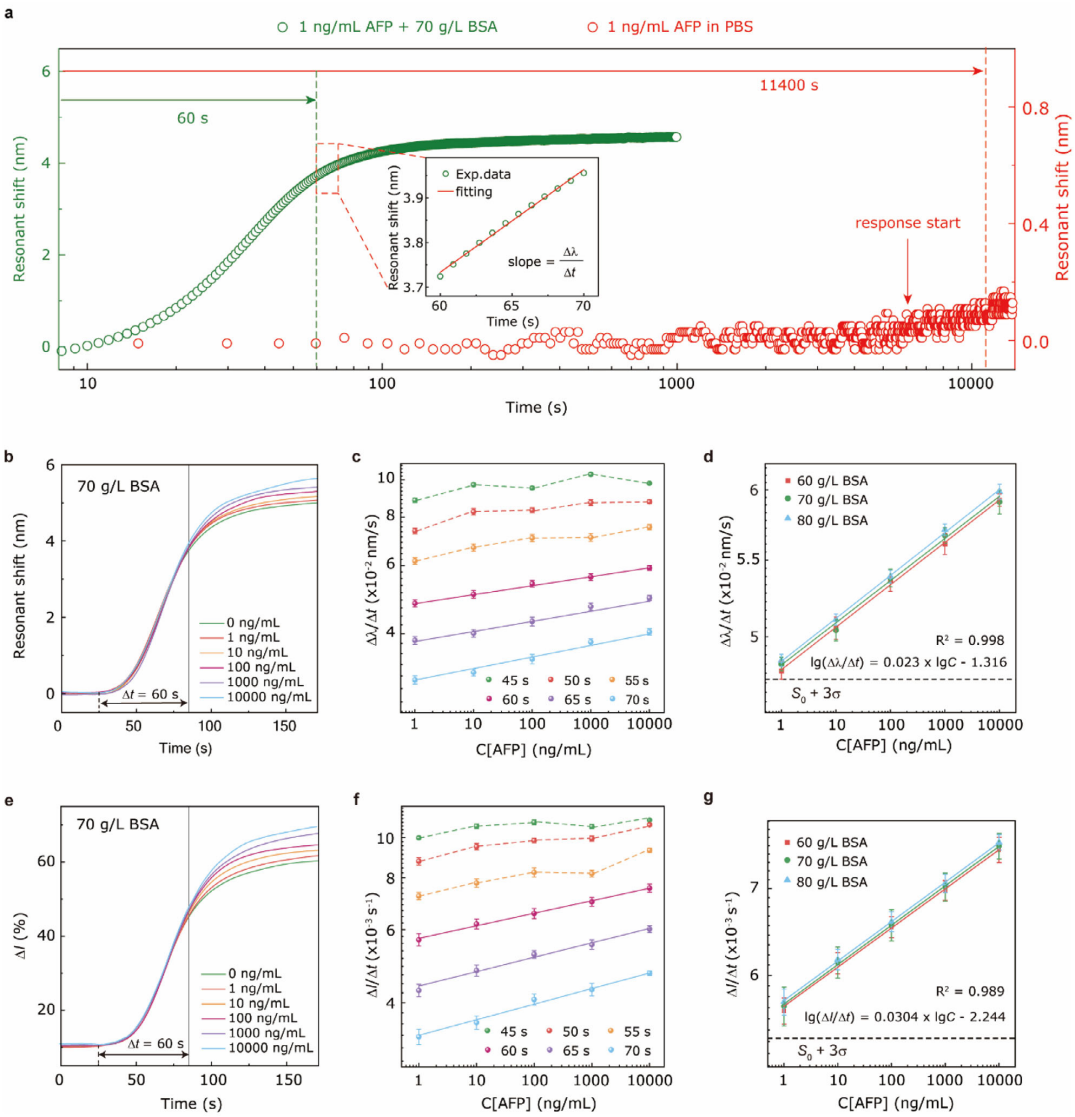
Accelerating tumor marker detection and eliminating background protein effect through introducing miscellaneous proteins and the IRA method. a) Comparison of the responses of a UGNA biosensor for 1 ng mL^−1^ AFP in PBS and 1 ng mL^−1^ AFP in 70 g L^−1^ BSA. b) Real‐time resonant shift curves of the AFP solutions with concentrations ranging from 0 to 10^4^ ng mL^−1^ in 70 g L^−1^ BSA. c) Response rates (Δλ/Δ*t*) as functions of AFP concentrations at different starting analysis points. d) Effect of the variation in BSA concentration on the response rates(Δλ/Δ*t*) at different AFP concentrations. e) Real‐time reflectance changing curves of the AFP solutions with various concentrations ranging from 0 to 10^4^ ng mL^−1^ in 70 g L^−1^ BSA at a fixed wavelength of 697.3 nm. f) Response rates (Δ*I*/Δ*t*) as functions of AFP concentrations at different starting analysis points. g) Effect of the variation in BSA concentration on the response rates (Δ*I*/Δ*t*) at different AFP concentrations. The error bars represent the standard deviations of three repeated measurements at each AFP concentration.

The clinical sample, such as human serum, is a crowded environment with a total protein concentration of ≈60–80 g L^−1^, which suggests the serum contains not only target analytes but also other high‐abundance proteins.^[^ ^42^ ^]^ This offers a great opportunity to verify the effect of the miscellaneous proteins on boosting the antigen‐antibody binding kinetics. BSA and HSA have 76 % structural similarity.^[^ ^53^, ^54^ ^]^ BSA is normally used as an alternative candidate to HSA because of its cheaper cost in biological experiments or biosensor development stages. The commercial Roche universal diluent (No. 07299001190) also uses animal serum protein. Therefore, we used BSA in the experiment process. We compared the measured response curves of 1 ng mL^−1^ AFP antigens in PBS with those in 70 g L^−1^ BSA, as shown in **Figure** **4**a. In the AFP/PBS solution, the onset of the response occurs after sample injection for ≈5500 s (red circles in Figure 4a). In contrast, in the AFP/BSA solution, a response was observed immediately after the solution was injected (within a few seconds, green circles in Figure 4a). Based on the IRA, we completed the concentration analysis from the slope (i.e., the response rate ∆𝜆/∆𝑡, the inset in Figure 4a) in a period of 60 to 70 s after the response began. This total detection time (≈70 s) for the AFP/BSA solution is 163‐fold faster than the time required to reach the equilibrium state (≈11400 s) for the AFP/PBS solution. We further investigated the dependence of the response rate (∆𝜆/∆𝑡) on the AFP concentrations. The response curves for a series of AFP solutions (containing 70 g L^−1^ BSA) with concentrations ranging from 0 to 10000 ng mL^−1^ are presented in Figure 4b. It is noteworthy that the response curves for each AFP concentration exhibit a nearly identical trend within the first 60 s, suggesting that the response is dominated by the change of the solution's RI in this period. To verify this, the initial analysis points were chosen to be 45, 50, 55, 60, 65, and 70 s after the response started, and the analysis time duration was set to be 10 s. Figure 4c reveals the relationships between ∆𝜆/∆𝑡 and the concentration of AFP antigen at various initial analysis points. For the 45–55 s initial analysis points, lg(∆𝜆/∆𝑡) are nonlinear with lg(*C*), which confirms that the response was affected by the bulk RI effect in this period. In contrast, starting from the initial analysis point of 60 s, linear relationships were observed between lg(∆𝜆/∆𝑡) and lg(*C*). Thus, 60 s was chosen as the starting point for the IRA in our experiments. Theoretically, the bulk reflective index transition occurs rapidly. Nevertheless, a certain degree of lag occurs in our microfluidic system due to the exchange between buffer and analyte solutions.

Nonspecific binding (NSB) remains a critical bottleneck in diagnostically relevant biosensing. Adsorbed interferents on the sensor surface not only obscure signals from trace‐concentration analytes but also block receptors, dramatically limiting the amounts of target analytes. Here, 60–80 g L^−1^ BSA solutions were employed to mimic the individual protein difference in the clinical sample, and their responses were measured using a UGNA biosensor (Figure S9, Supporting Information). The result indicates that the responses (∆𝜆) at the equilibrium state are different due to the variation in the bulk RI of the sample matrix. This suggests that it is challenging to directly obtain the analyte concentrations from the responses at the equilibrium state in practical biosensing. One effective method to circumvent this effect is rinsing to remove the unbounded molecules and reversible NSB. However, the rinsing process is generally time‐consuming and inefficient, making it unsuitable for developing a POCT strategy. We further investigated the effect of background protein variations (60, 70, and 80 g L^−1^ BSA) on IRA‐based AFP detections, as shown in Figure 4d. Surprisingly, the fitted lg(∆𝜆/Δ*t*)–lg(*C*) curves at different BSA concentrations are reasonably consistent. In addition, the black dashed line in Figure 4d represents the response signal *S*_0_ of the blank samples (i.e., 0 ng mL^−1^ AFP antigen in BSA solutions) plus three times its standard deviation ($3\sigma_{S_{0}}$). Considering the variation in background protein for each blank sample, both *S*_0_ and $3\sigma_{S_{0}}$ were calculated from three blank samples with different BSA concentrations of 60, 70, and 80 g L^−1^, where three repeated measurements were made at each BSA concentration. Accordingly, the LOD of 0.39 ng mL^−1^ and a dynamic linear range of 1–10^4^ ng mL^−1^ were achieved, even in the presence of nonspecific proteins at concentrations 10^5^–10^8^ times higher than the analyte. The miscellaneous‐protein‐mixed scheme combined with the IRA method significantly reduces total detection time while eliminating background protein interference. This advancement establishes a foundation for developing rapid diagnostic biosensing protocols. Furthermore, our tumor marker detection platform demonstrates an exceptionally broad linear range, surpassing both commercial chemiluminescence analyzers and reported plasmonic biomolecular platforms. The attribute is notably advantageous for the monitoring of treatment in late‐stage cancer patients, who frequently exhibit elevated tumor marker levels that surpass the typical linear range. This technological advancement significantly reduces the need for re‐dilution in tumor marker testing for these patients. It also minimizes procedural errors, lowers costs, and delivers highly accurate results, facilitating effective treatment monitoring.

From the perspective of commercial portable POCT devices, it is indispensable to eliminate the dependence on spectrometers. Compared to wavelength monitoring, intensity monitoring at a specific wavelength can significantly reduce costs and enhance device integration. We monitored the real‐time reflectance at *λ* = 697.3 nm close to the resonant dip. The relationship between lg(Δ*I*/Δ*t*) and lg(*C*) was analyzed, as shown in Figure 4e,f. Similar to the monitoring of resonant shift, lg(Δ*I*/Δ*t*) also exhibited a linear dependence on lg(*C*) when 60 s was used as the starting analysis point (Figure 4f). Moreover, the response rates lg(Δ*I*/Δ*t*) of the AFP antigens across a concentration gradient at different BSA concentrations were nearly consistent (Figure 4g). This suggests that the intensity‐based IRA method can also eliminate the effect caused by individual difference of the background protein level, and achieve a capability for direct detection of serum samples without a rinsing step. In addition, the intensity‐based LOD was also determined to be 0.13 ng mL^−1^.

The human serum environment is highly complex, presenting numerous potential interferents. To evaluate the robustness of the UGNA biosensor, we systematically assessed the effects of common serum interferents, including hemoglobin and bilirubin. Comparative analyses of UGNA‐Δ*λ*/Δ*t* and UGNA‐Δ*I*/Δ*t* against the control group are presented in Figure S10 (Supporting Information), with recovery rates (the percentage of measured analyte relative to the actual amount) and coefficient of variation (CV) values detailed in Tables S3 and S4 (Supporting Information). The UGNA biosensor maintains CVs <6.86% and recovery rates of 100.77‐111.21% at 500 mg dL^−1^ free hemoglobin, demonstrating robust resistance to hemolysis. This concentration far exceeds established upper reference limits for free hemoglobin in healthy subjects and unselected outpatients (5 or 22 mg dL^−1^, respectively).^[^ ^55^ ^]^ However, increasing hemoglobin concentrations progressively affected spectral intensity, leading to greater analytical deviations. Importantly, Δ*I*/Δ*t* analysis exhibited significantly elevated CVs at 1000 and 1500 mg dL^−1^. Importantly, free hemoglobin levels exceeding 500 mg dL^−1^ are widely recognized as indicating severe hemolysis and are grounds for sample rejection in clinical practice.^[^ ^56^ ^]^ For bilirubin, at concentrations < 15 mg dL^−1^, Δ*λ*/Δ*t* results remained within acceptable limits. Due to bilirubin's pronounced impact on spectral intensity, Δ*I*/Δ*t* analysis was deemed unreliable under these conditions. Investigation of other interfering substances such as blood lipids, Fe^3+^, and Fe^2+^ will be conducted in future studies.

## 6 Analysis of Clinical Samples

**Figure 5:**
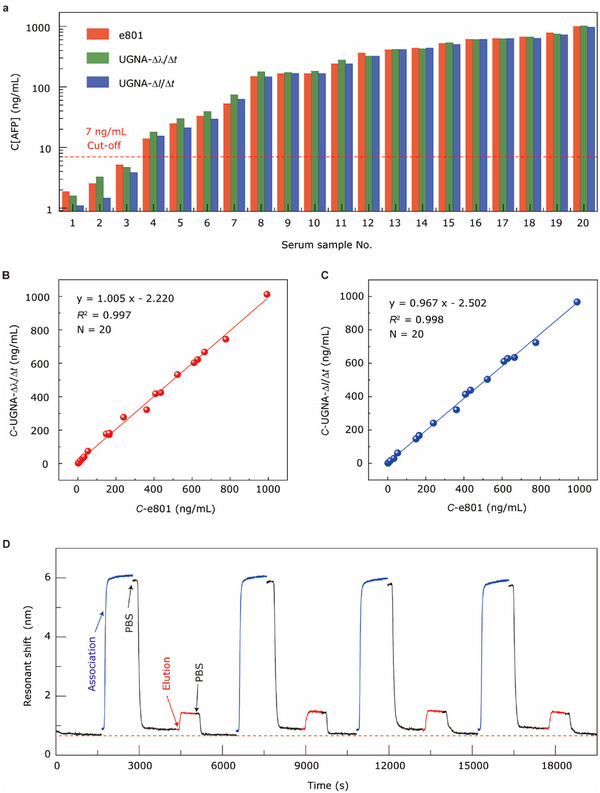
Clinical performance and regeneration of the UGNA biosensor. a) Comparison of the measured results for clinical samples between the commercial equipment (e801) and the UGNA biosensor combined with the IRA method. The red bars denote the results obtained from the e801, the green and blue bars represent the analyses by the UGNA biosensor at Δλ/Δ*t* and Δ*I*/Δ*t* modes, respectively. According to the Hygiene Industry Standard of China (WS/T 645.2–2018) and manufacturer's guidelines, the reference interval for AFP is ≤7 ng mL^−1^. This cut‐off value is marked with a dashed red line. b) Correlations between the experimental results obtained from the e801 and UGNA biosensor in the wavelength mode. c) Correlations between the experimental results obtained from the e801 and UGNA biosensor in the intensity mode. d) Resonant shift during the four cycles of AFP association and elution.

We collected 20 clinical serum samples with AFP antigen concentrations ranging from 1 to 1000 ng mL^−1^, and analyzed them using a UGNA biosensor combined with the IRA method. The Δλ/Δ*t* and Δ*I*/Δ*t* data were extracted at 60–70 s after analyte injection and used to calculate the AFP concentrations according to the calibration curves in Figure 4d,g. These results were compared with those obtained from the commercial Roche Cobas 8000 e801 analyzer (e801), as illustrated in **Figure** **5**a. The e801 utilizes electrochemiluminescence immunoassay (ECLIA) technology to detect the concentrations of analytes, and the entire detection process takes ≈18 min. The correlations between these two methods by linear fitting are presented in Figure 5b,c. The results show an excellent regressive relation (*R* ^2^ are 0.997 and 0.998, and slopes are 1.005 and 0.967 for the wavelength and intensity modes, respectively) between the ECLIA and our method, suggesting that our method shows high accuracy and holds great potential for clinical applications.

To enhance efficiency and reduce costs, we further developed a chip regeneration method enabling direct remeasurement after antigen elution. Figure 5d shows the resonant shift during the four cycles of association of 100 ng mL^−1^ AFP antigen in a 70 g L^−1^ BSA solution and subsequent elution by H_3_PO_4_/NaH_2_PO_4_ solution (The preparation steps are described in Methods), indicating that the anti‐AFP immobilized on the surface of the UGNA biosensor remains active after elution. This stability can be attributed to the stronger chemical bond between the antibody and the activated carboxyl groups on the UGNA surface, in contrast to the weaker antigen‐antibody interactions in a highly acidic environment (pH = 3). After four cycles of regeneration testing, SEM analysis confirmed that a large area of the UGNA structure remained intact, with a uniform distribution of antibody molecules across the nanogrooves (Figure S11, Supporting Information). The results demonstrate the robustness of the UGNA sensor, maintaining its structural integrity even after multiple cycles.

## 7 Discussion

In this work, our UGNA‐based nanoplasmonic biosensing technique offers a promising platform for label‐free, rapid, and quantitative protein detection in the serum samples over a broad linear range. The exceptionally high surface sensitivity and narrow resonant linewidth were simultaneously achieved by the coupling between the Fabry‐Pérot mode and Wood's anomaly mode in the UGNA. In addition, through the implementation of the miscellaneous protein scheme, the binding kinetics of the target analytes was immensely enhanced due to the suppression of the non‐specific consumption in the inlet tube. In conjunction with the IRA method, the total detection time was further reduced to ≈1 min, and the dynamic linear range was over 1–10^4^ ng mL^−1^, superior to mainstream commercial chemiluminescence analyzers. Moreover, the IRA method was demonstrated to eliminate the background protein effect and show excellent accuracy. The measured results of clinical samples were reasonably consistent with e801, showing a great potential value in clinical analysis. To accommodate a wider range of more complex clinical samples, we will continue to conduct in‐depth studies on interference analysis in the future, aiming to enhance UGNA biosensor's resistance to interference. Additionally, due to the superb sensing performance in the intensity mode under normal incidence, the miniaturization and multiplexing capabilities of our UGNA biosensor could be further enhanced by integrating it into a simple reflective imaging system. Finally, the deep learning‐based image recognition methods can introduce new inference tools to expedite our platform as a high‐throughput POCT biosensor for diagnostically relevant biosensing applications.

## Conflict of Interest

The authors declare no conflict of interest.

## Supporting information



Supporting Information

## Data Availability

Research data are not shared.
