# Supplementary material for: A Promising Point‐of‐Care Testing Strategy: Ultrasmooth Gold Nanogroove Arrays Biosensor Combined with Initial Rate Analysis
Source: Adv Sci (Weinh). 2025 Jul 22;12(36):e03056. doi: 10.1002/advs.202503056 (PMC12462996; doi:10.1002/advs.202503056)
Supplement: Supplementary file 1 — Supporting Information [file ADVS-12-e03056-s001.docx]

**A promising point-of-care testing strategy: ultrasmooth gold nanogroove arrays biosensor combined with initial rate analysis**

Yi Liu^1,*^, Shan Xing^2,*^, Zhan Si^1^, Kai He^1^, Manchun Zheng^1^, Yang Shen^1^, Chongjun Jin^1^

^1^State Key Laboratory of Optoelectronic Materials and Technologies, School of Materials Science and Engineering, Sun Yat-Sen University, No.132, East Outer Ring Road, Guangzhou Higher Education Mega Center, Guangzhou, Guangdong, 510006, China.

^2^Department of Clinical Laboratory, State Key Laboratory of Oncology in South China, Guangdong Provincial Clinical Research Center for Cancer, Sun Yat-sen University Cancer Center, 651 Dongfeng East Road, Guangzhou, Guangdong, 510060, China.

* These authors contributed equally to this work. Correspondence and requests for materials should be addressed to C.J.J. (email: jinchjun@mail.sysu.edu.cn) or to Y.S. (email: sheny33@mail.sysu.edu.cn).

1. **Methods**
   1. Materials and chemicals

Ethyl alcohol (99.5%) was purchased from Macklin. Gold target (99.999%) was obtained from Zhongnuo New Materials (Beijing) Technology Co., Ltd. Chromium target (99.95%) and SiO₂ target (99.99%) were purchased from JY Co., Ltd. UV-curing optical epoxy (NOA 61) was supplied by Norland Products Inc. Sulfuric acid (H_2_SO_4_, GR), hydrogen peroxide (H_2_O_2_, AR), phosphoric acid (H_3_PO_4_, 85%), and sodium dihydrogen phosphate (NaH_2_PO_4_, AR) were obtained from Guangzhou Chemical Reagent Factory. 11-Mercaptoundecanoic acid (MUA, 95%) and bovine serum albumin (BSA, ≥ 98%) were purchased from Sigma-Aldrich. Phosphate buffer solution (PBS) was sourced from ELGBio. N-(3-dimethylaminopropyl)-N'-ethylcarbodiimide hydrochloride (EDC), N-hydroxysuccinimide (NHS), and 4-morpholineethanesulfonic acid buffer (MES buffer, 0.5M, pH 5.0) were provided by Bioleaper. AFP antigen (Purified), anti-AFP McAb (Labeling), CEA antigen (Purified), anti-CEA McAb (Labeling), CA19-9 antigen, and anti-CA19-9 McAb (Coating & Labeling) were obtained from Shanghai Linc-Bio Science Co., Ltd. Polydimethylsiloxane (PDMS, Sylgard 184) was sourced from Dow Corning, and (tridecafluoro-1,1,2,2-tetrahydrooctyl)-1-trichlorosilane (TFOCS) was purchased from Sigma-Aldrich.

- 1. Equipment and instruments

Electron Beam Lithography (EBL, EBPG5000+, Raith) and Maskless Lithography System (uPG501) were used for patterning UGNA and micro-channel arrays, respectively. Deep Reactive Ion Etching (DRIE, Estrals, Oxford) was employed for Si template etching. Film deposition was performed using a Magnetron Sputtering system (VTC300, Micro-tech Co., Ltd., Shenyang). Scanning Electron Microscopy (SEM) (AURIGA, Zeiss) was used for observing microstructure topography. Fourier Transform Infrared Spectroscopy (FTIR) in Attenuated Total Reflection (ATR) mode (Frontier, PerkinElmer Inc.) was utilized to characterize the surface functional groups of the UGNA. Plasma treatment (TS-SY05, Dongxin Technology Co., Ltd.) was used for bonding pretreatment. UV radiation (wavelength 365 nm, 40 W) was applied to cure the UV-curing optical epoxy. An objective lens (UPlanSApo 4×, Olympus), supercontinuum laser (SC-Pro, YSL Photonics), spectrophotometer (iHR550, Horiba), EMCCD camera (DU970P-BVF, Andor), and syringe pump (RSP02-B, Biotaor) were used in the biosensor system.

- 1. Finite-difference time-domain simulations

Simulations were conducted using the FDTD solutions from Lumerical Solutions to generate the reflectance spectra and electric field intensity distributions of UGNAs. A linearly polarized plane wave normally illuminated to the grooves of the UGNA, with its polarization perpendicular to the grooves. The boundary conditions were set with periodic boundaries along the *x*-axis and perfectly matched layer (PML) boundaries along the *y*-axis. A mesh size of 1 nm was employed in the metal region. The dielectric functions of bulk gold were based on the experimental data from Johnson and Christy.

For the simulation of UGNAs' reflectance spectra: A series of UGNA simulated reflectance spectral structural parameters in Fig. 2d are all set based on the measured values from the SEM images (Fig. S6 and Table S2). The solid line is the reflectance spectrum before the self-assembly of MUA molecules, and surrounding medium is set as *n* = 1.333. The dashed line is the reflectance spectrum after the self-assembly of MUA molecules, specifically, a molecular layer with thickness *h* = 1.7 nm and *n*_mol_ = 1.45 is set on the surface of UGNA, and the surrounding medium is still set as *n* = 1.333.

- 1. Fabrication of UGNA biosensors

1. Fabrication of UGNA

The schematic diagram of the UGNA preparation using the template-stripped method is shown in Fig. S1. PMMA was spin-coated onto a Si substrate, and after patterning with EBL and developed. DRIE was used to etch the sample, forming a Si template with a UGNA. A 300 nm Au thin film was then deposited by magnetron sputtering. Epoxy resin was applied on top of the Au film, followed by placing a clean glass slide over it and UV curing for 18 min. The Au film was transferred from the Si template to the glass slide, forming the UGNA sensing component.

1. Fabrication of PDMS microchannels array.

The fabrication process of the PDMS microchannels array is shown in Fig. S2. The photoresist AR-P 3740 was first spin-coated onto a silicon substrate, followed by photolithography to form a random micropore array with diameters ranging from 1~3 μm. DRIE was then used to transfer the pore pattern onto the silicon substrate. Subsequently, AZ 2035 negative photoresist was spin-coated onto the as-etched substrate, and exposure and etching were performed to form a grating structure with a period of 40 μm, a duty cycle of 25%, and a depth of 5 μm. Another layer of AZ 2035 was spin-coated, exposed, and etched to create protruding structures with a height of 10 μm, thus completing the fabrication of the microfluidic channel array template.

Subsequently, the template surface was treated with a release agent (TFOCS) in a vacuum desiccator. Then, PDMS main agent and curing agent mixing (ratio of 10:1) was poured onto the template, degassed in a vacuum desiccator, and cured at 70 °C for 2 h to generate a PDMS microchannels array. The as-prepared PDMS microchannels were perforated with two holes (0.7 mm in diameter) at both ends of the groove, which served as an inlet and outlet for the microfluidic channel, respectively.

1. Integration of microfluidic-based UGNA sensor

After depositing 5 nm of chromium and 20 nm of SiO_2_ on the non-structural region of the UGNA prepared in step a, the as-deposited UGNA and the PDMS microchannel fabricated in step b were both treated with O_2_ plasma at 80 W for 1 min, as shown in Fig. S3. The two treated surfaces were then bonded together, followed by post-heating at 70 ℃ for 3 min to enhance the bonding strength of the PDMS/SiO_2_ interface, forming the microfluidic-based UGNA sensing platform.

- 1. Detection system based on UGNA biosensors

The schematic diagram of the UGNA-based detection system is shown in Fig. S8. The whole system consists of an optical detection system, a UGNA microfluidic sensing chip, and an injector. In the optical detection system, a supercontinuum laser beam was collimated and focused onto the sample surface by an objective lens, resulting in a spot size of 100 μm in diameter. The linearly polarized light generated by a Glan-Taylor prism was oriented perpendicular to the nanogrooves' direction. The reflected light from the sample surface was transmitted to a spectrophotometer and recorded by an EMCCD camera. All reflectance spectra were corrected using the reflectance spectrum of a silver mirror, which served only as a reference. Tygon tubing was inserted into the microfluidic channel's inlet and outlet, with the inlet connected to a syringe. The syringe was mounted on a syringe pump, with the sample flow rate set to 1 mL/h throughout all the injection procedures.

- 1. **Tumor marker detection in PBS**

UGNA was placed on the test stage, with the syringe pump maintained at a constant flow rate of 1 mL/h. Initially, PBS was injected into the microfluidic channel for spectral acquisition. Then, a (40 mM EDC & 10 mM NHS)/MES solution was injected for 15 min to activate the carboxylic terminal groups. The channel was washed with PBS for 15 min, followed by the injection of 100 µg/mL anti-AFP for 50 min for immobilization, and then washed again for 15 min with PBS. In the next step, 10 g/L BSA (with PBS as the solvent) was injected for 40 min to block the empty sites, followed by a 15 min wash with PBS. Finally, a series of antigen solutions with varying concentrations were injected. The reflection spectrum and the corresponding dip shift were monitored continuously. The antigen concentrations were chosen to be 1, 10, 100, 200, 400, 600, 800, 1000, and 10000 ng/mL. When testing with other antigens, the anti-AFP was replaced with the corresponding antibody at the same concentration.

For the specificity analysis of the UGNA biosensor, the sensor was functionalized individually with 100 µg/mL of anti-AFP, anti-CEA, and anti-CA19-9. The tested samples included 100 ng/mL of AFP, CEA, and 100 U/mL of CA19-9, a mixed antigen (containing 100 ng/mL each of AFP, CEA, and 100 U/mL of CA19-9), and PBS without any antigens, serving as a negative control.

- 1. **Tumor marker detection in** miscellaneous proteins

As described in Step 6, the anti-AFP was immobilized on the chip and blocked, followed by the injection of a series of solutions containing miscellaneous proteins into the channel. Reflectance spectra were then collected. The AFP solutions containing miscellaneous proteins were prepared as follows: BSA solutions with concentrations of 60, 70, and 80 g/L were prepared using PBS. These BSA solutions were then used as solvents to prepare AFP solutions with concentrations of 1, 10, 100, 1000 and 10000 ng/mL.

**1.8 Evaluation of the effects of common serum interferents**

As described in Step 6, after immobilizing the anti-AFP and blocking, interferent samples were injected, and the reflectance spectra were collected. With hemoglobin as the interferent, analyte AFP concentrations were set at 100 ng/mL and 1000 ng/mL. Hemoglobin was tested at three concentrations: 500 mg/dL, 1000 mg/dL, and 1500 mg/dL. With bilirubin as the interferent, analyte AFP concentrations were set at 100 ng/mL and 1000 ng/mL. Bilirubin was tested at three concentrations: 5 mg/dL, 10 mg/dL, and 15 mg/dL.

- 1. **Detection of Clinical Samples**

As described in Step 6, after immobilizing the anti-AFP and blocking, serum samples were injected, and the reflectance spectra were collected. To compare the detection results with those obtained from commercial equipment, the same clinical samples were analyzed by the Cobas e801 analyzer, where the AFP concentration in the serum was quantitatively measured through electrochemiluminescence (ECILA) signals generated by ruthenium (Ru) labeling.

1.10 Regeneration of UGNA biosensor chip

After the antigen analyte was injected into the analysis system, the channel was flushed with PBS to remove any excess analyte solution. Subsequently, an elution buffer was introduced and allowed to incubate for 15 min. The channel was then washed again with PBS to remove the dissociated antigen and any remaining elution buffer. The elution solution was prepared by dissolving 3.1202 g of NaH_2_PO_4_ and 167 μl of 85% H_3_PO_4_ in 100 mL of deionized water.

**1.11 Collection of** serum samples and ethics approval

Serum samples from 20 patients were collected at Sun Yat-sen University Cancer Center (SYSUCC) between January 1, 2024, and August 31, 2024. The acquisition of serum follows the routine procedures in SYSUCC. The Institutional Review Board of SYSUCC approved this study (B2024-429-01).

1. **Comparison of the key performance metrics for experimentally demonstrated plasmonic biomolecular detection platforms**


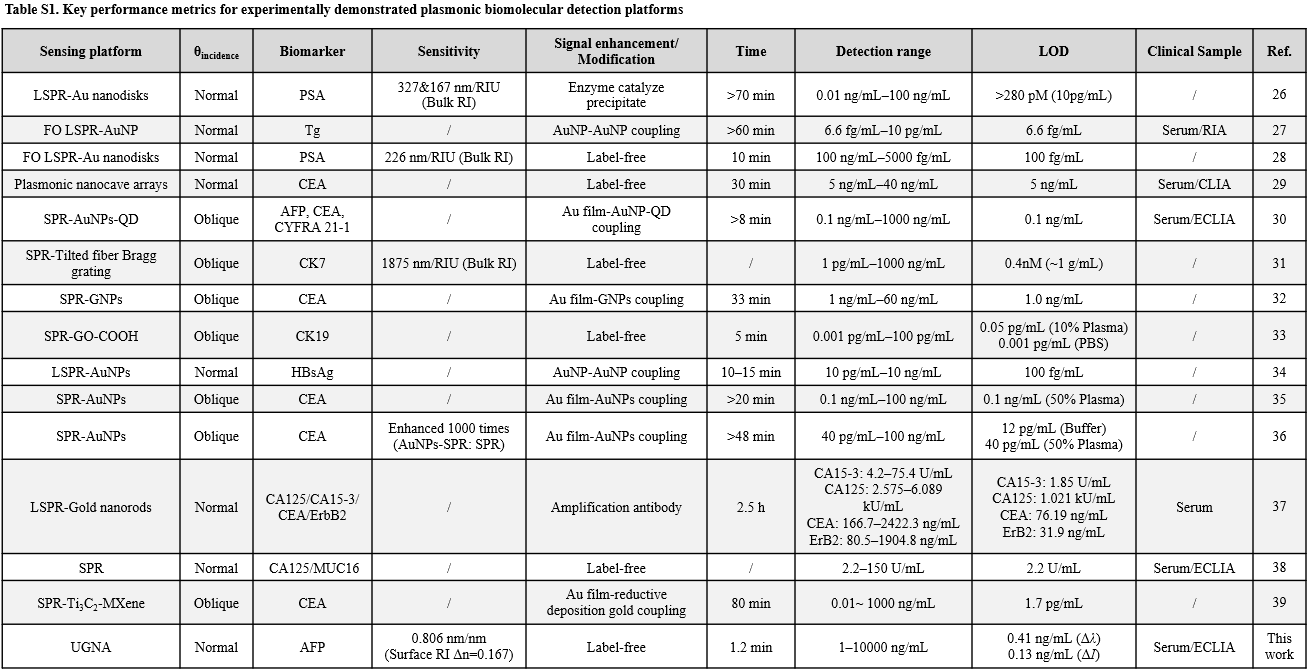


1. **Schematic diagram of the fabrication process of the UGNAs using the template-stripped method.**


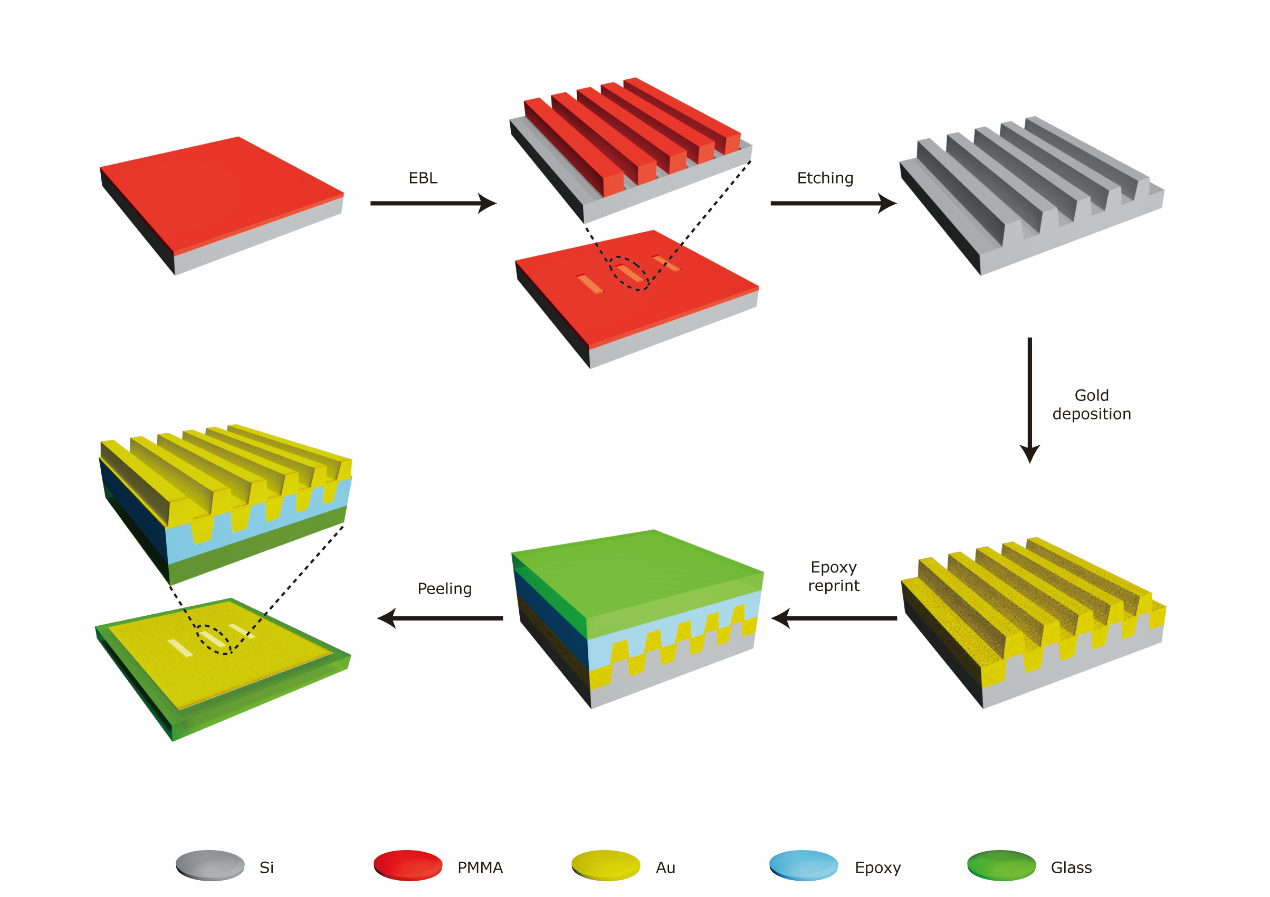


**Figure S1. Schematic diagram of the fabrication process of the UGNAs using the template-stripped method.** First, a Si template with a UGNA array is prepared by patterning with standard electron beam lithography (EBL) and deep reactive ion etching (DRIE). After depositing approximately 300 nm of Au film *via* magnetron sputtering, the pattern of the silicon template is transferred onto a glass substrate, leveraging the higher adhesion strength of Au to the epoxy resin compared to its adhesion to the Si template. This process ultimately results in the formation of the UGNA biosensing component.

1. **Schematic illustration of the fabrication process of the PDMS microchannels array.**


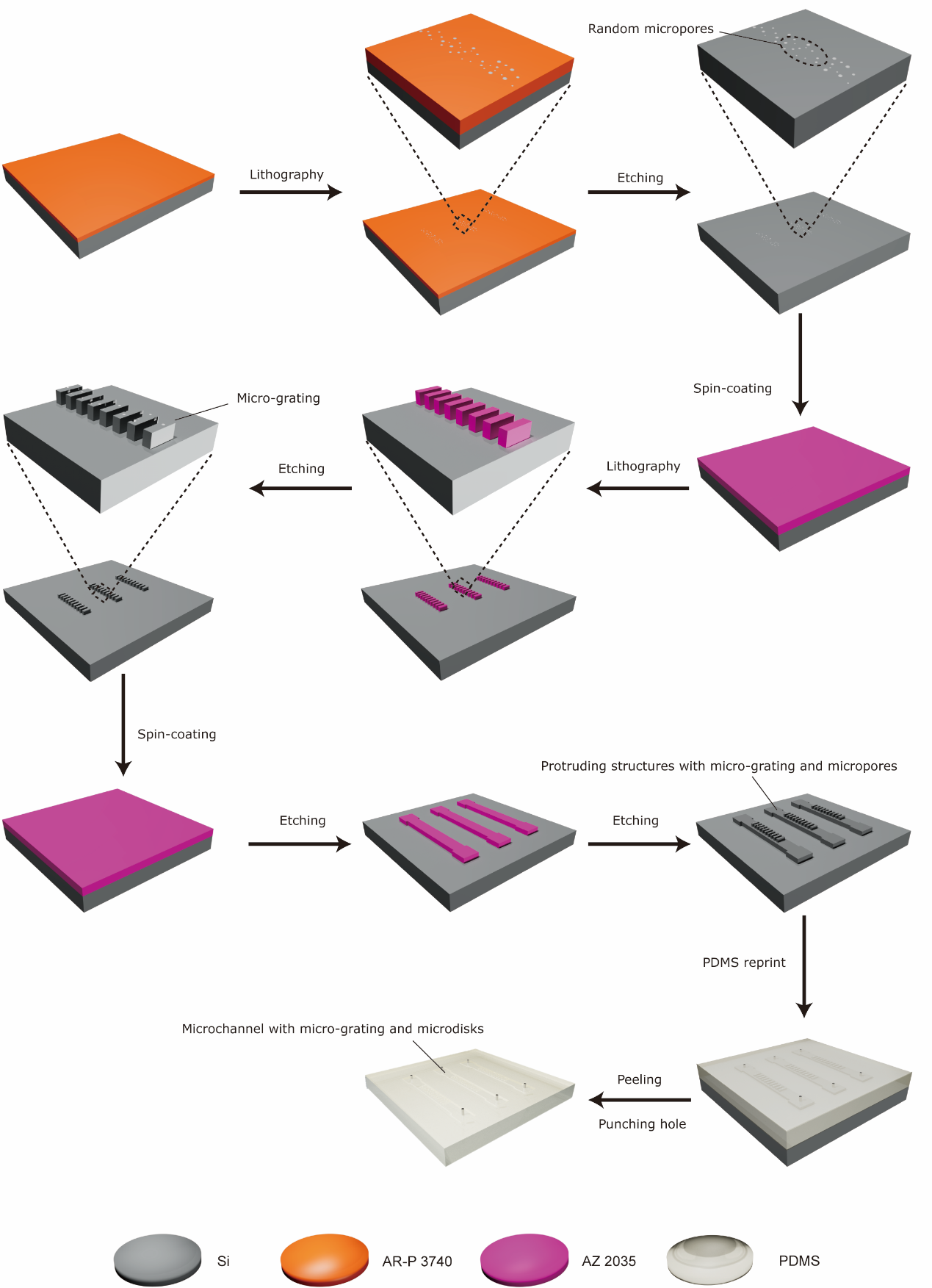


**Figure S2. Schematic illustration of the fabrication process of the microchannels array.** Preparation of microfluidic channel array structure template: An irregular hole array was first fabricated on a Si substrate using a maskless lithography device and DRIE. Subsequently, a micro-scale grating scale was prepared in the region of the holes array through lithography and etching. Finally, raised structures were fabricated at the designated positions using lithography and etching once more. Casting of the PDMS microfluidic channel: The PDMS fluid was prepared with a base-to-curing agent ratio of 10:1 and cast onto the template treated with a release agent. Following vacuum degassing, the PDMS was cured at 70 ℃ for 2 h, followed by demolding and punching.

1. **Diagram of the integration of a microfluidic UGNA-based sensing platform.**


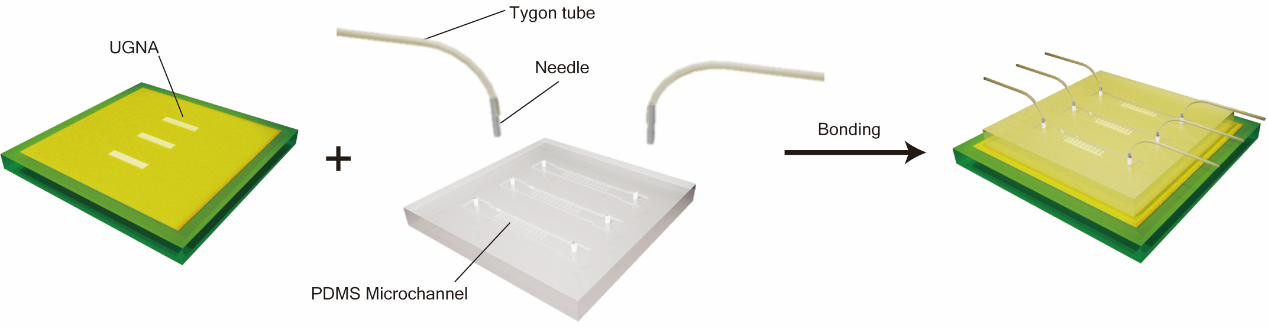


**Figure S3. Diagram of the integration of a microfluidic UGNA-based sensing platform.** After depositing 5 nm of Cr and 20 nm of SiO_2_ on the non-structural region of the UGNA biosensing component prepared in Step 2, the component and the PDMS microchannels fabricated in Step 3 were treated with O_2_ plasma and subsequently bonded together to form the UGNA sensing platform.

1. **Real-time responses of the AFP antigens with various concentrations in PBS.**


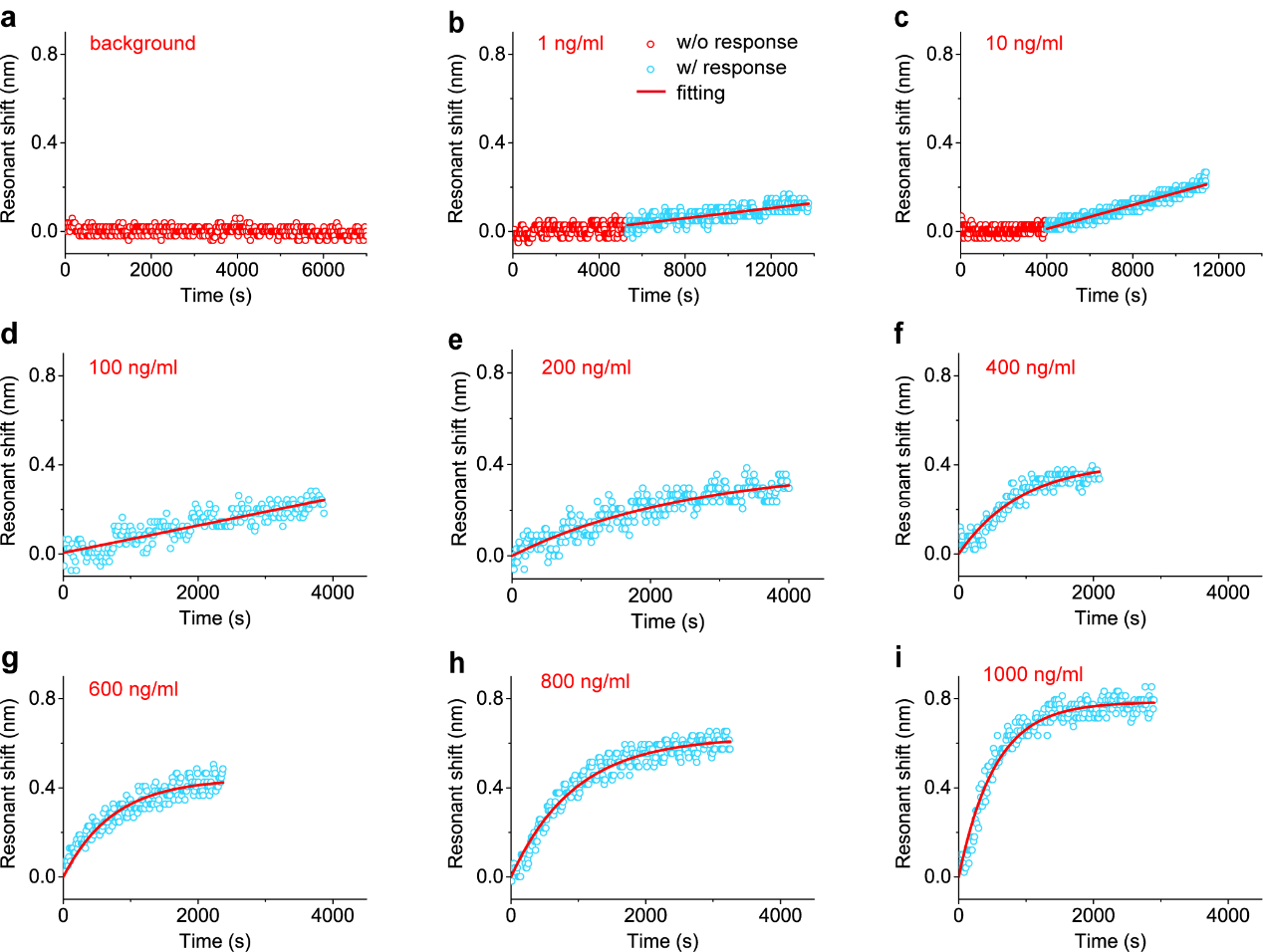


**Figure S4. Real-time responses of the AFP antigens with various concentrations in PBS.** At high concentrations, the binding of AFP to the sensor surface initially exhibits rapid growth and reaches equilibrium after a certain period. However, at low concentrations, an initial response delay is observed. For instance, for an AFP antigen concentration of 1 ng/mL, the response only begins after 5000 s, and the time taken to reach equilibrium can be as long as three hours.

1. **Optimization of UGNA biosensor sensing performance through geometric modulation.**


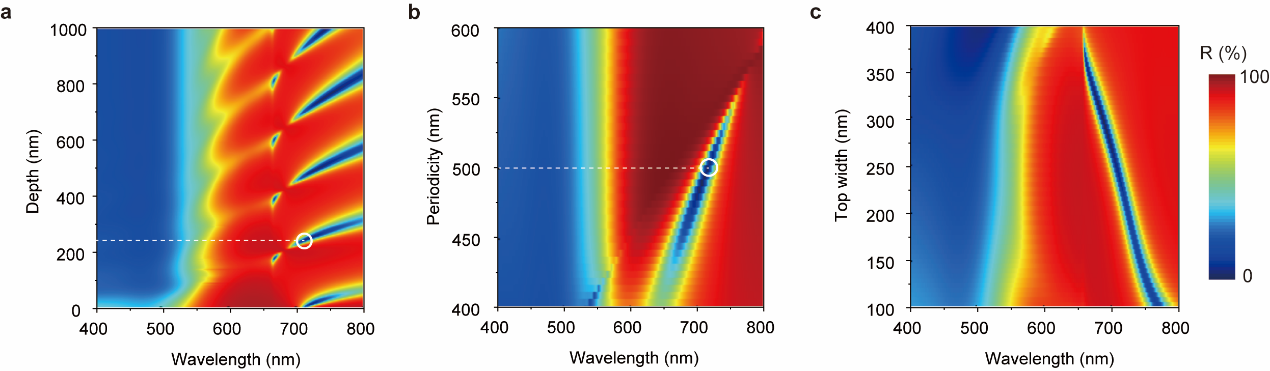


**Figure S5. Optimization of UGNA biosensor sensing performance through geometric modulation. a,** Simulated depth-modulated reflectance of the UGNAs, with parameters set at *a* =500 nm, *w*_1_ = 260 nm, *w*_2_ = 120 nm, for the depths ranging from 5 to 1000 nm. White circles correspond to h = 245 nm. **b,** Simulated reflectance spectra of the UGNAs as a function of the periodicity, with the white circles corresponding to a = 500 nm. **c,** Simulated reflectance spectra with width modulation of the UGNAs where *a* = 500 nm, *h* = 245 nm, for a top width of the groove varied from 100 to 400 nm. The reflectance intensity is nearly 0% over a broad modulation range.

1. **SEM topography of the UGNAs with various top widths ranging from 270 to 340 nm prepared by the template stripping method.**


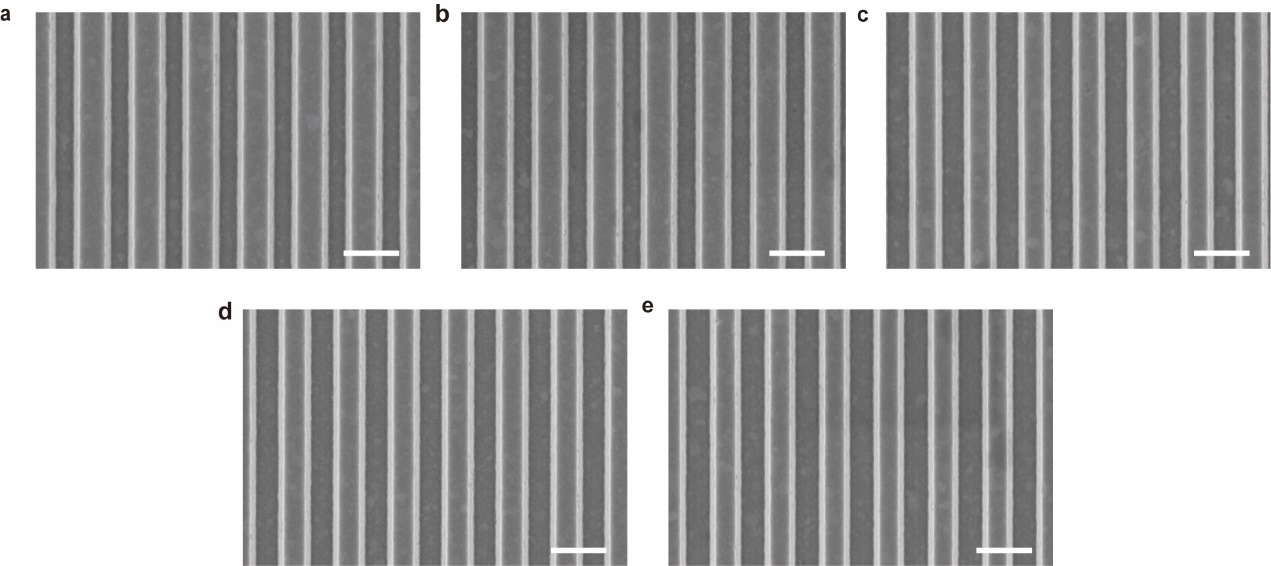


**Figure S6. SEM topography of the UGNAs with various top widths ranging from 280 to 340 nm prepared by the template stripping method.** The scale bars are 500 nm.

**Table S2. The structure parameters were measured from Figure S6 a-e.**

| Parameter | a | b | c | d | e |
| --- | --- | --- | --- | --- | --- |
| *a* | 500 | 500 | 500 | 500 | 500 |
| *h* | 245 | 245 | 245 | 245 | 245 |
| *w*_1_ | 280 | 295 | 310 | 320 | 340 |
| *w_2_* | 130 | 160 | 180 | 195 | 225 |

1. **FTIR analysis of a UGNA self-assembled with MUA.**


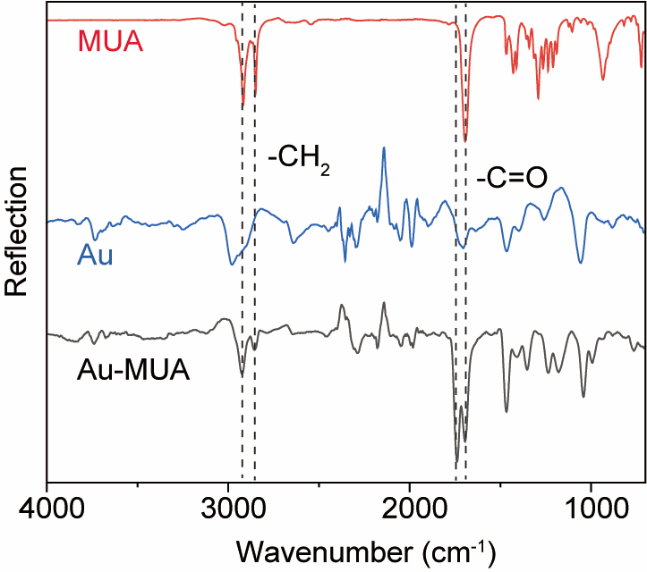


**Figure S7. FTIR analysis of a UGNA self-assembled with MUA.** We found several distinct strong peaks in the MUA spectrum (red): The strong peak at around 2900 cm-1 is characteristic of the C-H stretching vibrations in the long alkyl chain of MUA (-CH2-). The strong peak at around 1700 cm-1 is due to the carbonyl (C=O) stretching vibration of the carboxylic acid group (-COOH). The UGNA-MUA spectrum (black) shows a clear presence of the -CH2 peak around 2900 cm-1 and the C=O peak around 1700 cm-1. These peaks are more prominent in the UGNA-MUA spectrum compared to the UGNA spectrum (blue) alone. This suggests that MUA has been successfully attached to the UGNA surface.

1. **Schematic diagram of the whole UGNA biosensor system.**


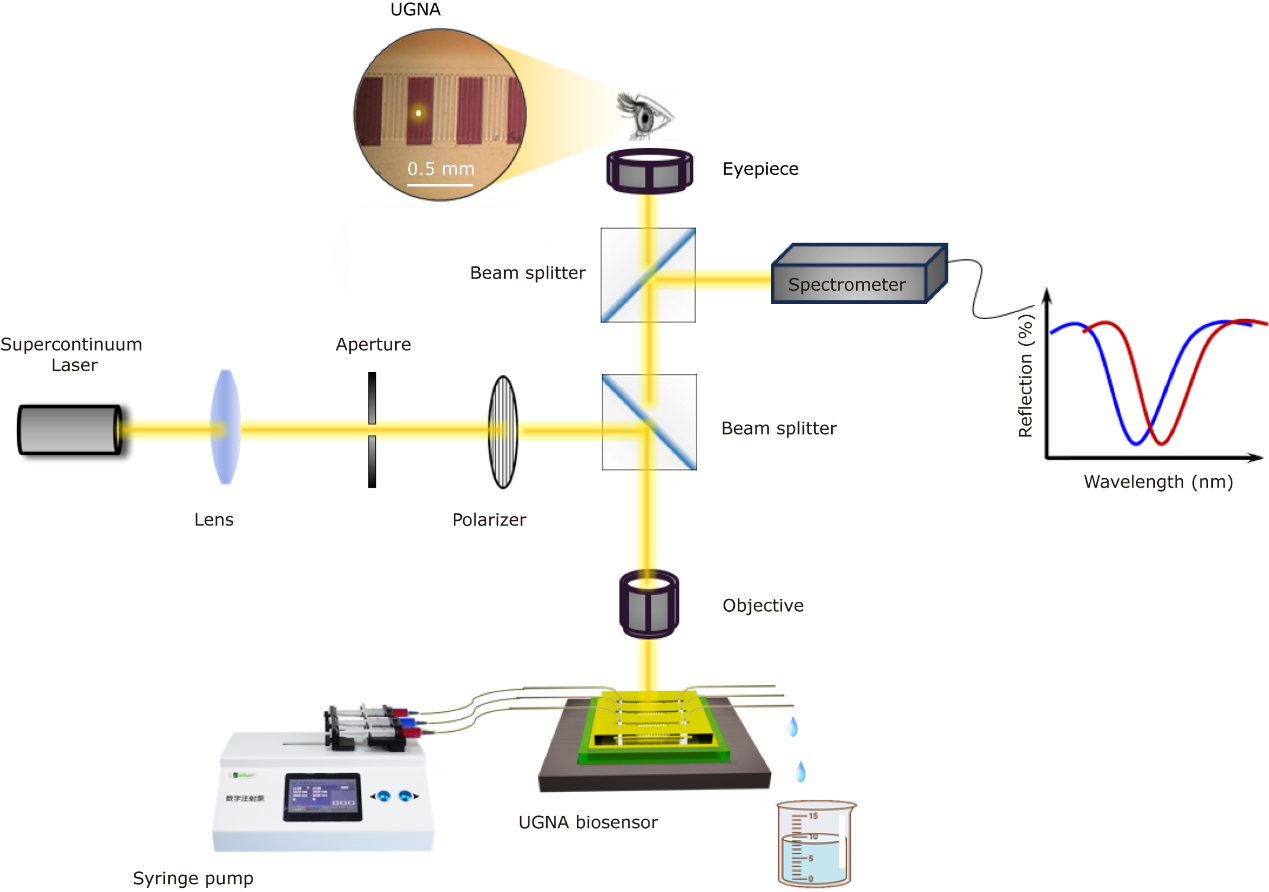


**Figure S8. Schematic diagram of the whole UGNA biosensor system.** A normally incident UGNA biosensor was integrated, comprising a sensing component (including a UGNA chip and microfluidic channel), a syringe pump, a light source, a detector, as well as some optical components used to control and adjust the propagation, intensity, polarization, and other characteristics of light.

1. **Real-time response curves of a UGNA biosensor for BSA solutions with different concentrations**

**
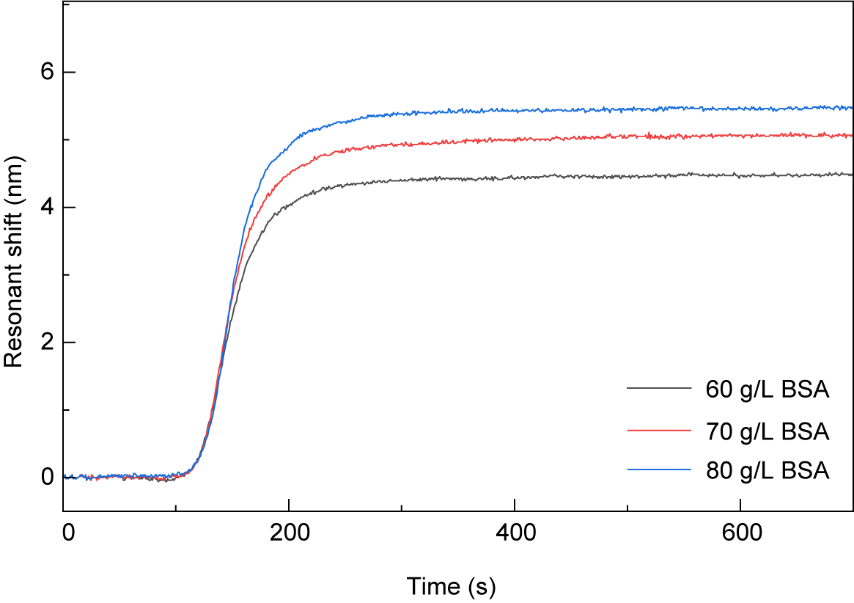
**

**Figure S9 Real**-**time response curves of a UGNA biosensor for BSA solutions with different concentrations.** Red, green, and blue curves represent 60 g/L, 70 g/L, and 80 g/L BSA in PBS, respectively.

1. **Analysis of interfering substances on UGNA biosensor.**


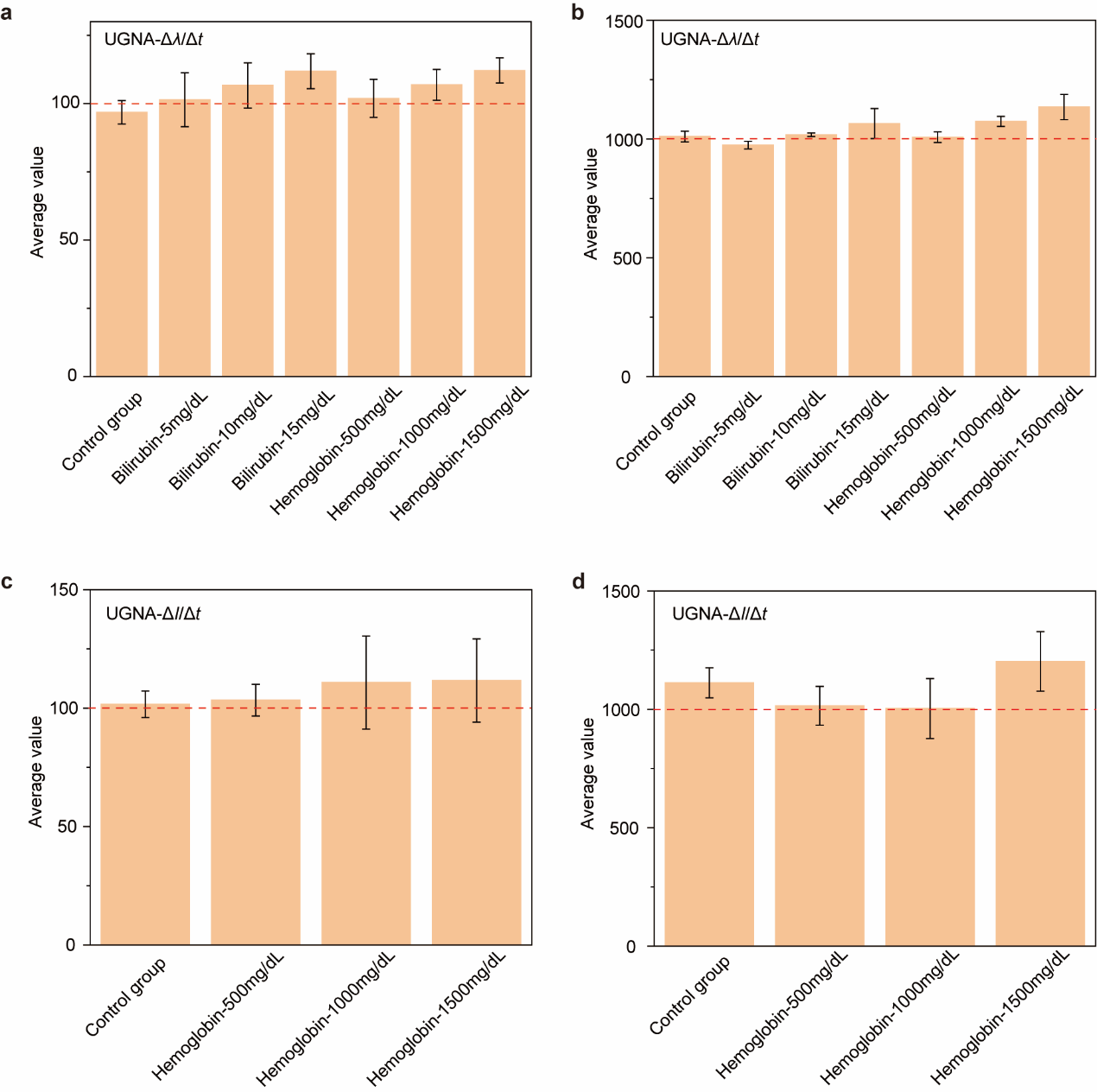


**Figure S10. The influence of common interfering substances in serum samples. a, b,** UGNA-Δ*λ*/Δ*t* detection results of 100 ng/mL and 1000 ng/mL AFP sample added different concentration bilirubin and hemoglobin, respectively. **c, d,** UGNA-Δ*I*/Δ*t* detection results of 100 ng/mL and 1000 ng/mL AFP sample added different concentration hemoglobin, respectively.

**Table S3. UGNA-Δ*λ*/Δ*t* detection results of recovery rate and CV of interfering substances**


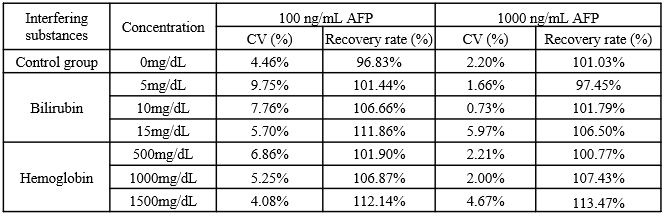


**Table S4. UGNA-Δ*λ*/Δ*I* detection results of recovery rate and CV of interfering substances**


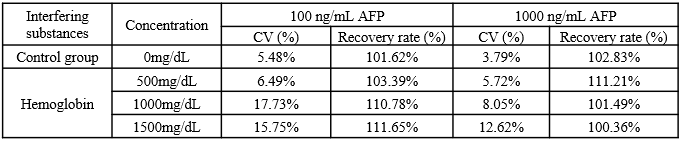


1. **SEM image of a UGNA after five cycles of AFP association and elution.**


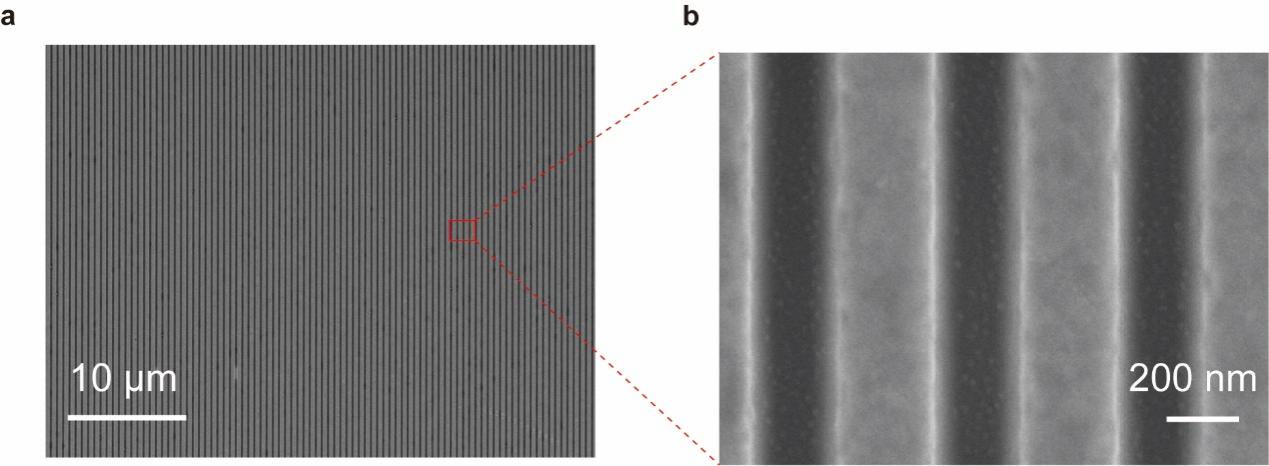


**Figure S11. SEM image of a UGNA after five cycles of AFP association and elution. a,** A large-scale SEM image of the UGNA, the UGNA structure remained intact. **b,** a local magnified view of (a) revealing the uniform distribution of anti-AFP molecules.
